# Phenotype and management of chronic obstructive pulmonary disease patients in general population in China: a nationally cross-sectional study

**DOI:** 10.1038/s41533-021-00243-x

**Published:** 2021-06-01

**Authors:** Heling Bao, Guohua Jia, Shu Cong, Wanlu Sun, Jing Fan, Ning Wang, Yajing Feng, Baohua Wang, Jeffrey L. Curtis, Linhong Wang, Liwen Fang, Yahong Chen

**Affiliations:** 1 National Center for Chronic and Non-communicable Disease Control and Prevention, Chinese Center for Disease Control and Prevention, 27 Nanwei Road, Xicheng District, Beijing, China; 2 Department of Maternal and Child Health, School of Public Health, Peking University, 38 Xueyuan Road, Haidian District, Beijing, China; 3 Department of Pulmonary and Critical Care Medicine, Peking University Third Hospital, 49 Huayuan North Road, Haidian District, Beijing, China; 4 Department of Internal Medicine, University of Michigan Medical School, 150 W. Medical Center Drive, Ann Arbor, MI USA; 5 Medicine Service, VA Ann Arbor Healthcare System, 2215 Fuller Road, Ann Arbor, MI USA

**Keywords:** Chronic obstructive pulmonary disease, Epidemiology

## Abstract

This study aims to investigate the characteristics of the phenotype and management of chronic obstructive pulmonary disease (COPD) patients in the general population in China. We analyzed spirometry-confirmed COPD patients who were identified from a population-based, nationally representative sample in China. All participants were measured with airflow limitation severity based on post-bronchodilator FEV_1_ percent predicted, bronchodilator responsiveness, exacerbation history, and respiratory symptoms. Among a total of 9134 COPD patients, 90.3% were non-exacerbators, 2.9% were frequent exacerbators without chronic bronchitis, 2.0% were frequent exacerbators with chronic bronchitis, and 4.8% were asthma-COPD overlap. Less than 5% of non-exacerbators ever had pulmonary function testing performed. The utilization rate of inhaled medication in non-exacerbators, exacerbators without chronic bronchitis, exacerbators with chronic bronchitis, and asthma-COPD overlap was 1.4, 23.5, 29.5, and 19.4%, respectively. A comprehensive strategy for the management of COPD patients based on phenotype in primary care is urgently needed.

## Introduction

Chronic obstructive pulmonary disease (COPD) is a globally public-health problem, with an estimation of 299.4 million prevalent cases and 3.2 million deaths per year worldwide^1,2^. In China, the COPD prevalence is 8.6% among adults with a projected 99.9 million patients^3,4^. COPD management greatly challenge the health-care systems, especially in low- and middle-income countries.

Early diagnosis and appropriate management of COPD patients in primary care play an important role in reducing the risk of adverse outcomes and improving quality of life, but this issue is paid not enough attention previously^5^. Furthermore, individualized management protocol based on specific COPD phenotype has been advocated instead of a “one size fits all” pattern^6,7^. Phenotypic strategies based on symptoms, response to therapy, and risk of disease progression are essential to direct the identification and management of COPD patients in routine practice^8^. However, great variations in terms of epidemiological characteristics and management protocols within ethnicity or nation complicates this goal. Although COPD phenotype distribution associated with clinically meaningful outcomes have been identified in clinical-based studies^9–11^, the issue is less known for COPD patients in general population in China.

Using data from a nationally representative, cross-sectional study in China^4^, we aimed to investigate the epidemiological and clinical characteristics, and management of COPD patients by phenotype in general population, according to a predefined criterion as well as Global Initiative for Chronic Obstructive Lung Disease (GOLD) classification.

## Results

### Participants

**Fig. 1:**
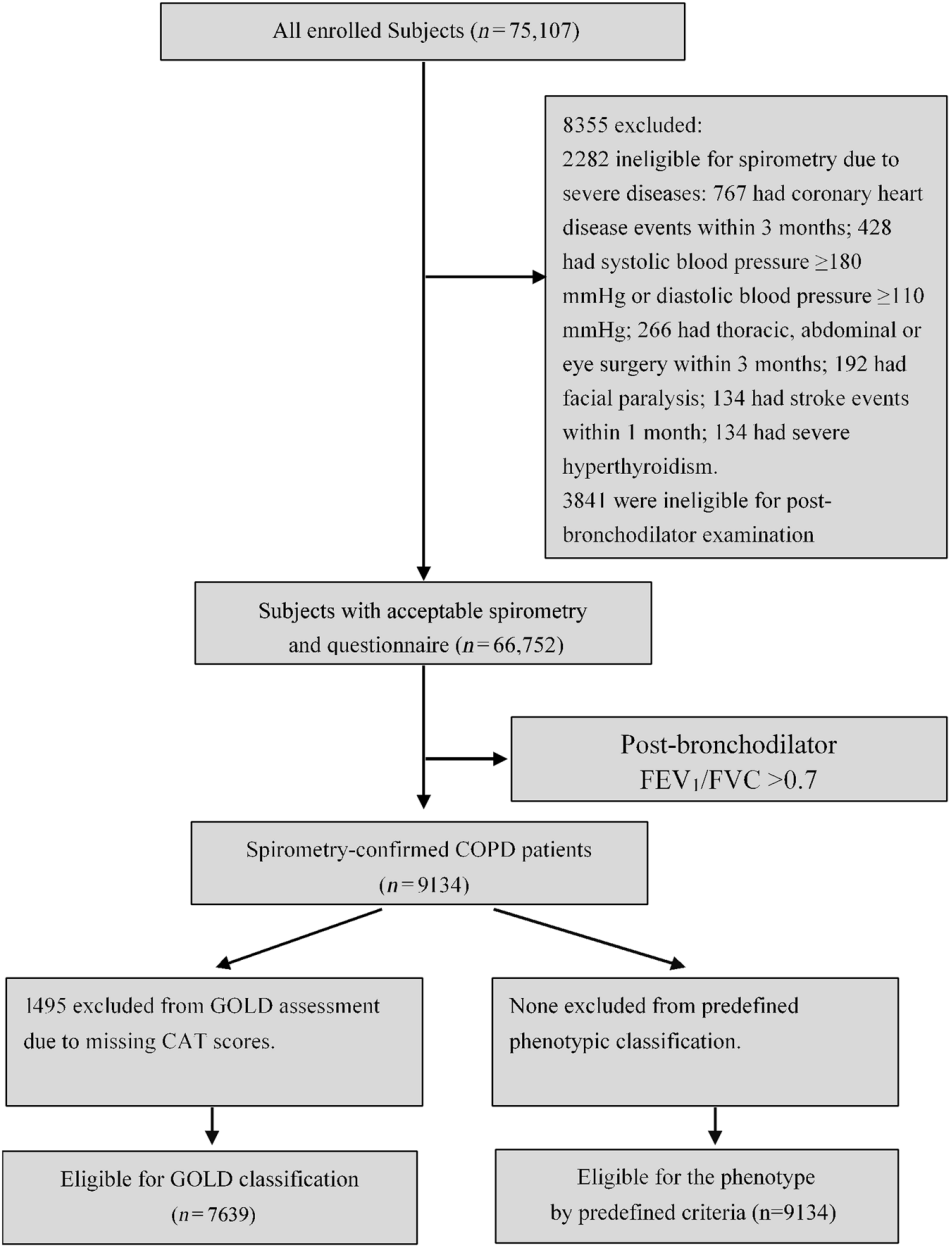
Study flow-diagram in the study: analysis of chronic obstructive pulmonary disease phenotype. Abbreviations: COPD, chronic obstructive pulmonary disease; FEV_1_, forced expiratory volume in one second; FVC, forced vital capacity; GOLD, Global Initiative for Chronic Obstructive Lung Disease; CAT, COPD assessment tool.

Of the 75,107 participants in the survey, 8355 participants were excluded due to severe diseases or ineligible spirometry, and 57,618 with a FEV_1_/FVC ≥0.7 were excluded (Fig. 1). Finally, the study detected 9134 spirometry-confirmed COPD patients aged ≥40 years, of whom all were available for classification of predefined phenotype and 7639 for GOLD classification. The mean age was 61.3 years (SD 9.6), 3326 were never-smokers, 246 were ever diagnosed as asthma, and 1132 patients were obesity (Table 1).

**Table 1:** Characteristics of patients with chronic obstructive pulmonary diseases according to phenotype.

	All patients	Phenotype				*P* value
		NON-AE (a)	AE-CB (b)	AE NON-CB (c)	ACO (d)	
Subjects, No. (%)	9134	8248 (90.3)	183 (2.0)	264 (2.9)	439 (4.8)	
Sex, No. (%), male	6635 (72.6)	6040 (73.2)^d^	141 (77.0)^c,d^	173 (65.5)^b^	281 (64.0)^a,b^	<0.001
Age, mean (SD), years	61.3 (9.6)	61.3 (9.6)^b,c,d^	64.6 (8.9)^a,d^	65.4 (9.4)^a,d^	58.8 (9.2)^a,b,c^	<0.001
Rural residence, No. (%), rural	5257 (57.6)	4762 (57.7)	114 (62.3)	150 (56.8)	231 (52.6)	0.27
Education Level, No. (%), primary or lower	5217 (57.1)	4691 (56.9)^b,c^	123 (67.2)^a,d^	177 (67.0)^a,d^	226 (51.5)^b,c^	<0.001
Smoking status, No. (%)						
Never smoker	3326 (36.5)	2977 (36.2)^d^	56 (30.8)^d^	102 (38.6)	191 (43.6)^a,b^	0.005
Former smoker	1445 (15.8)	1236 (15.0)^b,c^	45 (24.7)^a^	85 (32.2)^a,d^	79 (18.0)^c^	<0.001
Current smoker	4346 (47.7)	4020 (48.8)^c,d^	81 (44.5)^c^	77 (29.2)^a,b^	168 (38.4)^a^	<0.001
Smoking exposure, mean (SD), pack-years	22.0 (27.2)	22.0 (27.1)	27.5 (32.4)	20.8 (26.7)	19.3 (26.5)	0.08
Hospital admission for severe pulmonary disease in childhood, No. (%)	350 (3.8)	273 (3.3)^d^	10 (5.5)^d^	13 (4.9)^d^	54 (12.3)^a,b,c^	<0.001
Indoor to exposure to biomass pollutant, No. (%)	4602 (50.5)	4139 (50.2)	111 (61.0)	141 (53.4)	211 (48.2)	0.10
Exposure to dust or chemicals in workplace, No. (%)	4550 (49.9)	4068 (49.4)^b^	109 (59.6)^a^	144 (54.5)	229 (52.2)	0.02
Family history of respiratory disease, No. (%)	2847 (31.2)	2433 (26.6)^b,c,d^	91 (49.7)^a,c^	104 (39.4)^a,b,d^	219 (49.9)^a,c^	<0.001
Asthma, No. (%)	246 (2.7)	—	—	—	246 (56.0)	—
BMI, mean (SD), kg.m^−2^	24.0 (3.5)	24.0 (3.5)^b,c,d^	22.8 (3.8)^a,d^	23.4 (3.8)^a,d^	24.5 (3.9)^a,b,c^	<0.001
Obesity, No. (%)	1132 (12.4)	1015 (12.3) ^b,d^	18 (9.8) ^a,c,d^	34 (12.9) ^b,d^	65 (14.8) ^a,b,c^	<0.001
FEV_1_ % pred, mean (SD), %	81.6 (20.1)	83.2 (19.2)^b,c,d^	60.5 (23.3)^a,c,d^	66.6 (23.0)^a,b^	69.3 (19.4)^a,b^	<0.001
FVC % pred, mean (SD), %	103.8 (19.9)	104.9 (19.5)^b,c,d^	88.1 (21.5)^a,c,d^	93.5 (21.2)^a,b^	97.6 (19.4)^a,b^	<0.001
FEV_1_ / FVC, mean (SD), %	61.8 (8.3)	62.5 (7.6)^b,c,d^	53.0 (12.7)^a,d^	55.1 (11.8)^a^	56.1 (9.9)^a,b^	<0.001
GOLD Grade, No. (%)						
GOLD 1	5153 (56.4)	4907 (59.5)^b,c,d^	39 (21.3)^a^	80 (30.3)^a^	127 (28.9)^a^	<0.001
GOLD 2	3313 (36.3)	2886 (35.0)^d^	74 (40.4)^d^	113 (42.8)^d^	240 (54.7)^a,b,c^	<0.001
GOLD 3	590 (6.5)	412 (5.0)^b,c,d^	54 (29.5)^a,d^	59 (22.3)^a^	65 (14.8)^a,b^	<0.001
GOLD 4	78 (0.9)	43 (0.5)^b,c,d^	16 (8.7)^a,d^	12 (4.5)^a,d^	7 (1.6) ^a,b,c^	<0.001
CAT Score, mean (SD)	9.8 (7.3)	9.2 (6.8)^b,c,d^	17.9 (8.8)^a,c^	13.6 (7.4)^a,b^	15.6 (8.0)^a^	<0.001
mMRC Score, mean (SD)	0.2 (0.6)	0.2 (0.5)^b,c,d^	1.1 (1.1)^a,c,d^	0.8 (0.9)^a,b,d^	0.6 (0.9)^a,b,c^	<0.001
Exacerbation, mean (SD)						
Moderate AEs events per year	0.3 (1.9)	0.01 (0.1)^b,c,d^	4.8 (8.7)^a,d^	3.4 (3.4)^a,d^	1.1 (4.9)^a,b,c^	<0.001
Severe AEs events per year	0.1 (0.4)	0	0.9 (1.4)^a,d^	0.8 (0.9)^a,d^	0.3 (1.0)^a,b,c^	<0.001
Total AEs events per year	0.4 (2.1)	0.01 (0.1)^b,c,d^	5.7 (8.7)^a,d^	4.2 (3.5)^a,d^	1.3 (5.4)^a,b,c^	<0.001

*NON-AE* non-exacerbator, *AE CB* exacerbator with chronic bronchitis, *AE NON-CB* exacerbator without chronic bronchitis, *ACO* asthma-COPD overlap, *BMI* body mass index, *FEV1* forced expiratory volume in one second, *FVC* forced vital capacity, *GOLD* Global Initiative for Chronic Obstructive Lung Disease, *CAT* COPD assessment tool, *mMRC* Modified British Medical Research Council Questionnaire.

Data are presented as NO. (%) or mean (SD). ^a, b, c, d^ showed statistically significant difference between two phenotypes in pairwise comparisons, using Rao-Scott χ² test for category variables and Kruskal–Wallis test for continuous variables with Bonferroni-adjusted *P* value.

### Prevalence of COPD by phenotype

**Fig. 2:**
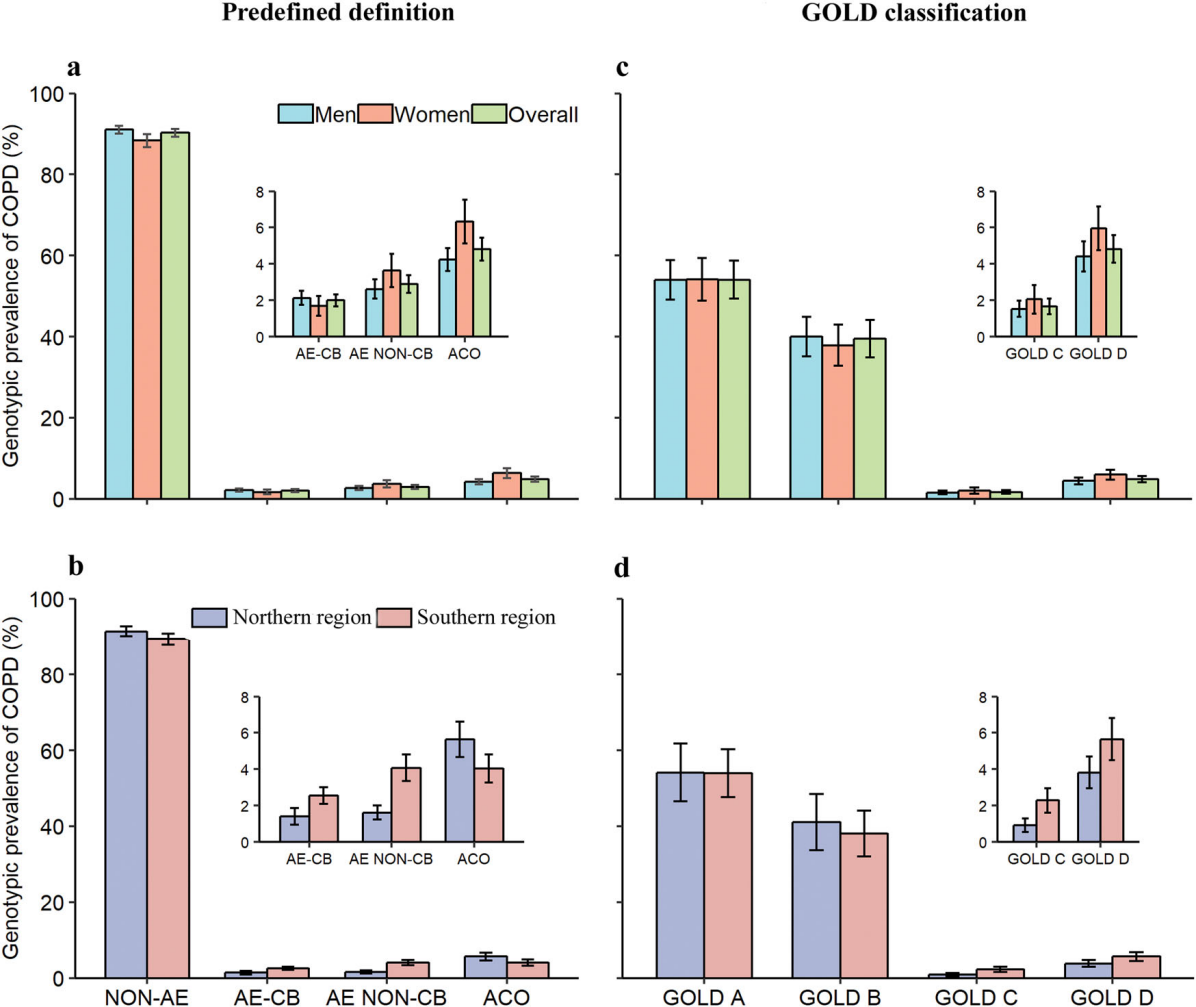
The prevalence of chronic obstructive pulmonary disease phenotype by sex and geographic locations. **a** COPD phenotype prevalence with 95% CI by sex, predefined definition. **b** COPD phenotype prevalence with 95% CI by geographic locations, predefined definition. **c** GOLD classification prevalence with 95% CI by sex, GOLD classification. **d** GOLD classification prevalence with 95% CI by geographic locations, GOLD classification. Error bars represented the standard error of the prevalence and were estimated by Taylor series variances estimation approach. Abbreviations: COPD, chronic obstructive pulmonary disease; GOLD, Global Initiative for Chronic Obstructive Lung Disease; NON-AE, non-exacerbator; AE-CB, exacerbator with chronic bronchitis; AE NON-CB, exacerbator without chronic bronchitis; ACO, asthma-COPD overlap.

The estimated COPD phenotype prevalence among COPD patients according to predefined definition were, NON-AE, 90.3% (95% CI, 89.3–91.3%), AE NON-CB, 2.9% (2.4–3.4%), AE-CB, 2.0% (1.7–2.3%), and ACO, 4.8% (4.2–5.4%), while the pattern according to GOLD was GOLD A, 54.1% (95% CI, 49.3–58.8%), GOLD B 39.5% (34.8–44.1%), GOLD C, 1.7% (1.2–2.1%), and GOLD D, 4.8% (4.1–5.6%) (Fig. 2). All NON-AE patients were in GOLDA through B group, and AE with or without CB were in GOLD C through D group. Of ACO patients, approximately 78% were in GOLD A through B, whereas 22% in GOLD C through D. (Supplementary Table 1). We also did a sensitivity analysis using the lower limit of normal to define COPD and this gave a similar pattern of phenotype prevalence (Supplementary Table 2).

Based on ~77.2 million COPD patients aged 40 years or old in China, we project 69.7 million NON-AE patients, 3.8 million with frequent exacerbations, 3.7 million ACO patients, whereas 35.5 million patients with high risk of exacerbation or more syndromes (B through D grades).

NON-AE was more prevalent in men than women (91.0 vs. 88.4%, *P* < 0.001), whereas ACO (4.2 vs. 6.3%, *P* < 0.001) was more common in women than men (Fig. 2). Both AE-CB (2.6 vs. 1.4%, *P* = 0.001) and AE NON-CB (4.1 vs. 1.6%, *P* < 0.001) were more prevalent in the south than that in the north, whereas the pattern was inverse for ACO (4.0 vs. 5.6%, *P* = 0.01). In contrast, we did not observe systematically patterns of the GOLD classifications in term of sex and geographic locations. We examined in greater detail the finding of geographic variation with respect to predefined phenotypes; and found that the geographic pattern was associated with regional-level temperature (Supplementary Tables 3 and 4).

### Symptom burden and comorbidity of COPD patients by phenotype

Prevalence of risk factors, symptom burden and airflow limitation severity significantly differed by predefined phenotypes and GOLD classifications (Table 1 and Supplementary Table 5). For example, current smoking was more prevalent in NON-AE (48.8%) and AE-CB (44.5%) than that in AE NON-CB (29.2%). Current smoking was more prevalent in GOLD A and B (49.0 and 50.7%) than that in GOLD C and D (31.5 and 34.1%). Among 439 ACO patients, 191 were never-smokers, most of whom were exposure to biomass or occupational pollutants (Supplementary Table 6). A history of severe pulmonary diseases in childhood was most frequent in ACO (12.3%) than that in other phenotypes.

AE-CB had the highest CAT or mMRC scores and the worst lung function as compared with NON-AE. Lung function and symptom burden were worse in patients with ACO as compared with NON-AE, but better than AE regardless of co-existing CB. The total exacerbation events were substantially higher in AE-CB (5.7 events·year^−1^) and AE NON-CB (4.2 events·year^−1^), and moderately higher in ACO (1.3 events·year^−1^) as compared with NON-AE (0.01 events·year^−1^). Similar systematic patterns in terms of symptom and airway limitation were also observed in GOLD classification.

Overall, 32.1% of COPD patients had comorbidities (Table 2 and Supplementary Table 7), and the common comorbidities among COPD patients were hypertension (21.3%), cerebrovascular disease (5.9%), coronary artery disease (5.7%), diabetes mellitus (5.3%), and osteoporosis (4.4%). AE-CB, AE NON-CB and ACO patients more often had comorbidities or multiple comorbidities as compared with NON-AE (*P* < 0.001). Similarly, the prevalence of comorbidities was higher in GOLD C and D as compared with GOLD A and B.

**Table 2:** Prevalence of comorbidities in patients with chronic obstructive pulmonary disease according to phenotype.

	All patients	Phenotype				*P* value
		NON-AE (a)	AE-CB (b)	AE NON-CB (c)	ACO (d)	
Comorbidities	2930 (32.1)	2550 (30.9)^b,c,d^	85 (46.4)^a^	121 (45.8)^a^	174 (39.6)^a^	<0.001
Solid tumor	66 (0.7)	10 (0.1)^b^	2 (1.1)^a^	0 (0)	0 (0)	—
Coronary artery disease	520 (5.7)	421 (5.1)^b,c,d^	22 (12.0)^a^	33 (12.5)^a^	44 (10.2)^a^	<0.001
Cerebrovascular disease	540 (5.9)	461 (5.6)^b,c^	21 (11.5)^a^	27 (10.2)^a^	31 (7.1)	0.003
Diabetes mellitus	485 (5.3)	434 (5.3)	12 (6.6)	16 (6.1)	23 (5.2)	0.81
Hypertension	1943 (21.3)	1723 (20.9)	49 (26.8)	66 (25.0)	105 (23.9)	0.06
Depression	45 (0.5)	38 (0.5)^b^	4 (2.2)^a,d^	2 (0.8)	1 (0.2)^b^	0.009
Osteoporosis	405 (4.4)	327 (4.0)^b,c^	18 (9.8)^a^	25 (9.5)^a^	35 (8.0)	<0.001
Comorbidity number						
0	6201 (67.9)	5695 (69.1)^b,c,d^	98 (53.6)^a^	143 (54.2)^a^	265 (60.4)^a^	<0.001
1–2	2716 (29.7)	2378 (28.8)^b,c,d^	71 (38.8)^a^	109 (41.3)^a^	158 (36.0)^a^	<0.001
≥3	214 (2.3)	172 (2.1)^b,c,d^	14 (7.7)^a,d^	12 (4.5)^a^	16 (3.6)^a,b^	<0.001

*NON-AE* non-exacerbator, *AE-CB* exacerbator with chronic bronchitis, *AE NON-CB* exacerbator without chronic bronchitis, *ACO* asthma-COPD overlap.

Note: Data are presented as NO. (%). ^a, b, c, d^ showed statistically significant difference between two phenotypes in pairwise comparisons, tested by Rao-Scott χ² test using Bonferroni-adjusted p value.

### Management of COPD patients

Overall, 5.9% of COPD patients ever had pulmonary function performed, 3.4% ever utilized inhaled medications and 2.5% ever had oxygen therapy (Table 3), whereas less than 1% of patients received long-acting or short-acting bronchodilators with more utilization of short-acting bronchodilators (0.9%) than long-acting bronchodilator (0.3%) or multiple therapies (0.4%). By comparison, 10.4% ever received non-inhaled medications, with most commonly antibiotics (5.5%), antitussives (5.3%), traditional Chinese medicines (3.5%), and theophylline (3.1%). Inhaled medication utilization was higher in AE-CB (29.5%), AE NON-CB (23.5%), and ACO (19.4%) as compared with NON-AE (1.4%; *P* < 0.001). Utilization of non-inhaled medications was also greater in AE-CB and AE NON-CB than in NON-AE or ACO (*P* < 0.001). The treatment using mono- or multiple corticosteroids-containing inhalers was notably low in ACO patients (2.5%).

**Table 3:** Treatment and prevention in patients with chronic obstructive pulmonary disease according to phenotype.

	All patients	Phenotype				*P* value
		NON-AE (a)	AE CB (b)	AE NON-CB (c)	ACO (d)	
**Inhaled or non-inhaled therapy**						
**Inhaled therapy**	314 (3.4)	113 (1.4)^b,c,d^	54 (29.5)^a,d^	62 (23.5)^a^	85 (19.4)^a^	<0.001
Mono-ICS	9 (0.1)	2 (0.02)^b,c,d^	1 (0.5)^a^	1 (0.4)^a^	5 (1.1)^a^	<0.001
Short-acting bronchodilators	84 (0.9)	35 (0.4)^b,c,d^	13 (7.1)^a^	10 (3.8)^a^	26 (5.9)^a^	<0.001
Long-acting bronchodilators	31 (0.3)	13 (0.2)^b,c,d^	3 (1.6)^a^	7 (2.7)^a^	8 (1.8)^a^	<0.001
Combined therapy ^e^	36 (0.4)	11 (0.1)^b,c,d^	6 (3.3)^a^	5 (1.9)^a^	14 (3.2)^a^	<0.001
Combined ICS ^e^	16 (0.2)	4 (0.1)^b,c,d^	2 (1.1)^a^	4 (1.5)^a^	6 (1.4)^a^	<0.001
Unknown pattern ^f^	154 (1.7)	52 (0.6)^b,c,d^	31 (16.9)^a,d^	39 (14.8)^a,d^	32 (7.3)^a,b,c^	<0.001
**Non-inhaled therapy**	952 (10.4)	506 (6.1)^b,c,d^	127 (69.4)^a,d^	159 (60.2)^a,d^	160 (36.4)^a,b,c^	<0.001
Theophylline	284 (3.1)	128 (1.6)^b,c,d^	44 (24.0)^a,c^	30 (11.4)^a,b^	82 (18.7)^a^	<0.001
Oral corticosteroids	47 (0.5)	16 (0.2)^b,c,d^	7 (3.8)^a^	12 (4.5)^a^	12 (2.7)^a^	<0.001
Expectorant	196 (2.1)	93 (1.1)^b,c,d^	42 (23.0)^a,c,d^	31 (11.7)^a,b^	30 (6.8)^a,b^	<0.001
Antioxidants	16 (0.2)	10 (0.1)^c^	1 (0.5)	4 (1.5)^a^	1 (0.2)	<0.001
Antitussive	486 (5.3)	257 (3.1)^b,c,d^	71 (38.8)^a,d^	72 (27.3)^a^	86 (19.6)^a,b^	<0.001
Antibiotics	502 (5.5)	268 (3.2)^b,c,d^	71 (38.8)^a,d^	81 (30.7)^a,d^	82 (18.7)^a,b,c^	<0.001
Traditional Chinese medicine	317 (3.5)	160 (1.9)^b,c,d^	41 (22.4)^a^	57 (21.6)^a^	59 (13.4)^a^	<0.001
Antiallergic medicine	65 (0.7)	23 (0.3)^b,c,d^	13 (7.1)^a^	9 (3.4)^a^	20 (4.6)^a^	<0.001
**PFT performed before**	540 (5.9)	395 (4.8)^b,c,d^	33 (18.0)^a^	26 (9.8)^a,d^	86 (19.6)^a,c^	<0.001
**Rehabilitation**	73 (0.8)	29 (0.4) ^b,c,d^	13 (7.1)^a^	9 (3.4)^a^	22 (5.0)^a^	<0.001
**Oxygen therapy**	229 (2.5)	66 (0.8)^b,c,d^	50 (27.3)^a,d^	62 (23.5)^a^	51 (11.6)^a,b^	<0.001
**Influenza vaccine**	320 (3.5)	263 (3.2)^d^	11 (6.0)	11 (4.2)	35 (8.0)^a^	<0.001
**Pneumococcal vaccine**	77 (0.8)	58 (0.7)^b,c^	4 (2.2)^a^	8 (3.0)^a^	7 (1.6)	<0.001

*NON-AE* non-exacerbator, *AE CB* exacerbator with chronic bronchitis, *AE NON-CB* exacerbator without chronic bronchitis, *ACO* asthma-COPD overlap, *ICS* inhaled corticosteroid, *PFT* pulmonary function testing.

Note: Data are presented as No. (%).

^a, b, c, d^ showed statistically significant difference between two phenotypes in pairwise comparisons, using Rao-Scott χ² test with Bonferroni-adjusted p value.

^e^ Combined ICS was contained in the combined therapy.

^f^ Patients do not know or remember the type of inhaled medications.

Similarly, the utilization rates of inhaled medication were relatively low in GOLD C (26.8%) and D (31.5%), however the proportion in GOLD A and B was greatly decreased (1.1 and 3.1%) (Supplementary Table 8). Additionally, the utilization of corticosteroids in patients who were classified as GOLD C or D and ACO was 10.3%, where the proportion in patients in GOLD A and B was only 1.6% (data no showed).

## Discussion

The population-based study provided comprehensive evidence on the phenotypic prevalence, epidemiological and clinical characteristics of COPD patients by phenotype in general population in China. The study estimated that 90% of COPD patients were non-exacerbators, and approximate 5% were frequent exacerbator with high-risk of poor outcomes. Furthermore, the study revealed that the proportions of COPD management with respect to diagnosis, inhaled medications, and risk factors control were substantially low in primary care.

The study displayed the phenotypic pattern of COPD patients by two different classifications: a predefined criterion and the GOLD classification. The GOLD classification is regarded as the best-available management and prediction tool in clinical practice^12,13^. Nonetheless, some studies noticed that the individual management should be tailed according to the risk and phenotype related to the clinical presentation of COPD^14,15^. We defined a phenotype algorithm according to the GesEPOC, which was first established in 2012 and adopted by some national guidelines^14,16^. In the GesEPOC, COPD patients were classified as non-exacerbators, asthma-COPD overlapped, and exacerbators with emphysema or chronic bronchitis. However, the emphysema was difficult to be confirmed in population-based study due to the cost of the chest computed tomography, and therefore AE NON-CB was used as alternative^9^. The diagnosis of ACO in a given patient included three elements: significant smoking exposure (more than 10 pack-years), irreversible airflow limitation, and asthma. But in our study, the smoking exposure was not an essential element in the ACO patients because the exposure to biomass fuel and occupational pollutants were also reported as main risk factors of COPD in China^15,17^.

Our results showed that most of the COPD patients in general population of China were in low-risk status with less or no symptoms and mild airway obstruction, consistent with other studies in primary care^18,19^. Compared with studies using similar definition of exacerbator, the AE prevalence in our study was similar with the PLATINO study in the Latin America (6% in 2009)^20^, but substantially lower than the POPE cohort in the central and eastern European (30% in 2014–2015)^9^. The ACO prevalence was similar with that in the USA and European countries (6% in 2012–2013)^21^, but lower than estimates from Latin America (13% in 2010)^20^ and Japan (9% in 2017)^22^. Correspondingly, the proportions of high-risk GOLD categories in our study were comparable to the COPDGene cohort (0.8% in GOLD C; 5% in GOLD D)^10^, but lower than the ECLIPSE (7 and 15%)^23^ and POPE (2 and 36%)^12^ cohorts. Under the similar definition, the discordant pattern may reflect the heterogeneity of population, different epidemiological characteristics of risk factors, diagnosis, and management of patients.

We observed a systematic pattern of the AE and ACO phenotypes by geographic locations, which might be associated with the difference in temperature and humidity between the south and north. It was well-known that the south was hotter and more humid than the north in China. Previous studies linked the excess risk of respiratory viral or bacterial infection to higher temperature and associated humidity with variations in exacerbation frequency^14,24^. In contrast, cold air exposure is related to bronchial hyperresponsiveness^25^. Further investigations of potential mechanisms (genetic susceptibility, respiratory infections, and lifestyle patterns) are needed, but the results imply the role of geographic location in the management of COPD patients.

Another important finding was that COPD patients within any phenotype were underdiagnosed or undertreated in China, inconsistent with other studies in western countries^26,27^. Long-acting bronchodilators alone or in combination were considered as the basis of pharmacological treatment of COPD^16,28^, however, in our study, less than 5% of patients prescribed inhaled medication and less than 30% of AE phenotype ever used inhaled medications. The underdiagnosis of COPD patients might contribute to the undertreatment. Furthermore, the utilization of short-acting bronchodilators was more common than long-acting bronchodilators, implying the goal of current treatment was preferred for the control of symptoms rather than improvement of quality of life and lung function. In contrast, the prevalence of conventional risk factors associated with exacerbation risk, e.g., current smoking^29,30^, underweight or obesity^27^, and indoor or occupational exposure^15,17^, were substantially high in each phenotype. Additionally, that less than 5% received influenza or pneumococcal vaccines, and less than 3% received rehabilitation or oxygen therapy, underscoring the opportunity for primary care intervention in China.

The ACO phenotype was worth of being independently identified in the management, and guidelines advocated the long-acting bronchodilators combined with ICS-containing medications in ACO treatment^16,31^. Our results showed the utilization of inhaled bronchodilators and ICS-containing medications among ACO patients in our survey (19 and 5%, respectively) compare unfavorably with other studies, such as Latin American data (49 and 23%, respectively)^18^. This may be associated with low proportion of ACO patients fell into the GOLD D group (20% of ACO) for which the ICS-containing medications were recommended under the GOLD classifications, but the exact reasons are complicated and still uncertain. Nonetheless, our results showed that the identification of ACO phenotype in management of COPD patients in primary care is of essential not regardless of using GOLD categories or other definitions.

Due to the nature of the study, most of the COPD patients in our study were non-exacerbators, who were underdiagnosed, less symptomatic or asymptomatic, and had mild airway obstruction, indicating the relatively low risk of poor outcomes. Furthermore, the proportion of never-smokers was relatively high (36%). Therefore, the data could not show the real-life treatment of hospital-based COPD patients^30,32^, but underlined the necessity of management for COPD in primary care, for example, early diagnosis of at-risk patients, smoking cessation, regular pharmacological treatment.

Findings suggested that a fixed ratio (<0.7) might lead to more frequent diagnosis in elderly population and less in younger^28^, which further affect the estimation of COPD phenotype in the study. Based on the spirometric parameters established for Chinese population, we did a sensitivity analysis using the lower limit of normal, and the proportion of NON-AE slightly increased without statistical significance. Nonetheless, careful analyses are necessary for this issue.

A strength of our study is the large amount of COPD patients from a representative sample enable us to analyze the phenotype prevalence and epidemiological characteristics of COPD patients from general population. Nonetheless, several limitations should be discussed. First, among 8355 excluded participants due to ineligible spirometry, 1084 reported physician-diagnosed COPD or COPD-related symptoms (data not showed), and therefore the cross-sectional study might underestimate the risk of exacerbation in the patients. Our estimates should be considered the minimum values for the high-risk classifications. Second, the inclusion of never-smokers in the ACO phenotype might introduce the misclassification bias in the estimation. Third, in our study, the obese patients accounted for 12% of COPD patients and the obesity state had impact on the measurement and estimation of spirometric parameters (i.e., FEV_1_, FVC, FEV_1_/FVC)^33^. This would affect the diagnosis of COPD and phenotypic characteristics. Final but not the least, the comorbidities and treatment were self-reported but not validated using systematic review of healthcare records.

In conclusions, most of the COPD patients in general population of China were non-exacerbators and approximately 5% were exacerbators. However, early diagnosis and standard treatment were obviously inadequate for each phenotype. A comprehensive strategy for the management of COPD patients based on phenotype in primary care is urgently needed.

## Methods

### Study design and population

This analysis derived from a previously reported nationally, population-based, cross-sectional study from December 2014 to December 2015^4^. Briefly, the study used a complex, multistage, probability sampling strategy, and included 75,107 Chinese citizens aged 40 years or older who had been living in their current residence for at least 6 months within the year. A total of 66,752 participants had acceptable post-bronchodilator spirometry examinations. The diagnosis of COPD was based on post-bronchodilator spirometry showing a ratio of forced expiratory volume in the first second (FEV_1_)/forced vital capacity (FVC) <0.7 per the Global Initiative for Chronic Obstructive Lung Disease (GOLD) 2018 guidelines^28^. The participants with an abnormal FEV1/FVC ratio were further examined by chest X-ray to exclude suspicious patients with tuberculosis, bronchiectasis, or tumor. In this study, we included spirometry-confirmed COPD patients with FEV_1_/FVC <0.7 for this study to reflect the real-world characteristics and management of COPD patients in general population in China.

### Measurement and definitions

For each participant, we collected demographic characteristics, respiratory symptoms, risk factors, medical history (i.e., frequency of hospital visit and admission, comorbidities, inhaled or non-inhaled medications, rehabilitation, oxygen therapies, and vaccines), the COPD assessment test (CAT), and the modified Medical Research Council (mMRC) dyspnea scale, through questionnaires. Spirometry was operated by trained technicians using the same brand of spirometer (MasterScreen Pneumo, Jaeger, Germany) according to the criteria of American Thoracic Society. Exacerbations were defined as hospital visits related to acute worsening of respiratory symptoms (increased dyspnea, cough, phlegm, and wheeze) resulting in treatment with antibiotics or oral corticosteroids (moderate exacerbation) or hospital admission (severe exacerbation)^29^. We divided patients into symptomatic and less symptomatic group based on CAT score (<10 and ≥10), as well as mMRC grade (0–1 and 2–4), respectively.

We defined patients as four phenotypes according to the Spanish COPD guidelines (GesEPOC) and related studies:^9,16,31^ patients with asthma-COPD overlap (ACO), non-exacerbators (NON-AE), frequent exacerbators with chronic bronchitis (AE-CB) and frequent exacerbators without chronic bronchitis (AE NON-CB). The ACO phenotype was defined as COPD patients who were diagnosed with asthma before 40 years or those who had a positive bronchial dilation test (comprising increases in FEV_1_ of both ≥ 12% and 200 mL) with asthma or allergy symptoms including wheezing or coughing due to exercise or exposure to cold air or pollutants. Patients who had at least one hospital admissions, two or more moderate exacerbations, or both during the past 12 months were classified as frequent exacerbators (AE). Patients with a maximum of one moderate exacerbation and no hospital admission during the past 12 months were classified as NON-AE. Chronic bronchitis (CB) was identified as sputum production with or without chronic cough more than three months per year during two continuous years. AE Patients with CB were recorded as AE-CB, whereas others were recorded as AE NON-CB. Additionally, we classified patients using the ABCD assessment tool of GOLD 2018 guidelines to describe the characteristics of COPD patients.

The severity of airflow limitation was measured by the absolute FEV_1_, the predicted FEV_1_ estimated using reference equations for Chinese population aged 4–80 years old^34^, and by the ratio of absolute FEV_1_/the predicted FEV_1_ (FEV_1_ % pred.). Also, we estimated the lower limited normal (LLN) of FEV_1_/FVC to define COPD patient as sensitivity analysis. Occupational exposure was defined as exposure to airborne dust or hazardous chemical gases in the workplace for at least 12 months. Indoor exposure to biomass was defined as household use of biomass fuels (e.g., wood, grass, crop residues, and animal dung) for cooking or heating. Smoking cessation defined as patients who had quitted smoking for at least 12 months. Comorbidities were defined as self-reported solid tumor, coronary heart disease, cerebrovascular disease, diabetes mellitus, hypertension, depression, and osteoporosis.

### Statistical analysis

We estimated the phenotypic prevalence with confidential intervals (95% CI) in the overall patients, in subgroups of sex, and geographic locations (north versus south). The phenotypic prevalence was defined as the proportion of patients classified as distinct phenotypes among all COPD patients. We characterized and compared the demographic, conventional risk factors, lung function, symptom burden, comorbidities, and management between/among phenotypes. One-way or multiple comparisons between/among phenotypes were conducted considering the complex sampling. We used analysis of variance (ANOVA) or Kruskal–Wallis test for continuous variables, and Rao-Scott χ² test for categorical variables for comparison. All hypothesis testing was judged at the 0.05 significance level (two-sided). Bonferroni correction for pairwise comparisons was used. All statistical analysis was carried out using SAS version 9.4 and using R software (version 3.5.4).

### Reporting summary

Further information on research design is available in the Nature Research Reporting Summary linked to this article.

## Supplementary information

Supplementary Information

Reporting Summary

## Data Availability

All data that support the findings of this study are included in this published article and its supplementary information files. The datasets are available on request from the corresponding author.

## Code availability

Available upon request.
