# Supplementary material for: Phenotype and management of chronic obstructive pulmonary disease patients in general population in China: a nationally cross-sectional study
Source: NPJ Prim Care Respir Med. 2021 Jun 1;31:32. doi: 10.1038/s41533-021-00243-x (PMC8169915; doi:10.1038/s41533-021-00243-x)
Supplement: Supplementary file 1 — Supplementary Information [file 41533_2021_243_MOESM1_ESM.pdf]

Supplementary Table 1. Distribution of COPD patients with phenotype by GOLD classifications.

GOLD assessment	All patients	Phenotype			
		NON-AE (a)	AE CB (b)	AE NON-CB (c)	ACO (d)
**GOLD assessment (CAT)** [e]					
GOLD A	4129 (54·1)	4043 (59·2)[d]	-	-	86 (21·9)[a]
GOLD B	3015 (39·5)	2795 (40·9)[d]	-	-	220 (56·0)[a]
GOLD C	127 (1·7)	-	35 (20·8)[c,d]	82 (34·2)[b,d]	10 (2·5)[b,c]
GOLD D	368 (4·8)	-	133 (79·2)[c,d]	158 (65·8)[b,d]	77 (19·6)[b,c]
**GOLD assessment (mMRC)** [f]					
GOLD A	8269 (90·9)	7962 (96·8)[d]	-	-	307 (70·1)[a]
GOLD B	295 (3·2)	259 (3·2)[d]	-	-	36 (8·2)[a]
GOLD C	419 (4·6)	-	128 (71·1)[c,d]	217 (82·8)[b,d]	74 (16·9)[b,c]
GOLD D	118 (1·3)	-	52 (28·9)[c,d]	45 (17·2)[b,d]	21 (4·8)[b,c]

Abbreviations: NON-AE, non-exacerbator; AE-CB, exacerbator with chronic bronchitis; AE NON-CB, exacerbator without chronic bronchitis; ACO, asthma-COPD overlap; GOLD, Global Initiative for Chronic Obstructive Lung Disease; CAT, COPD assessment tool; mMRC, Modified British Medical Research Council Questionnaire.

Note: Data are presented as No. (%). [a, b, c, d] indicate statistically significant difference between two phenotypes in pairwise comparisons, tested by Rao-Scott χ^2 test using Bonferroni-adjusted p values.

[e] 1495 COPD patients were excluded for GOLD category by CAT due to missing CAT scores.

[f] 33 COPD patients were excluded for GOLD category by mMRC due to missing mMRC scores.

Supplementary Table 2. Phenotypic characteristics of COPD patients who were defined by the lower limited normal value of FEV1/FVC.

	All patients	Phenotype				*P* value
		NON-AE (a)	AE-CB (b)	AE NON-CB (c)	ACO (d)	
**Subjects**, No. (%)	9593	8700 (90.7)	180 (1.9)	257 (2.7)	456 (4.8)	
**Sex**, No. (%), male	6697 (69.8)	6109 (70.2)	138 (76.7)	165 (64.2)	285 (62.5)^{a,b}	**<0.001**
**Age**, mean (SD), years	58.6 (10.1)	58.4 (10.1)^{b,d}	63.4 (9.1)^{a}	64.4 (10.0)	58.0 (9.4)^{a}	**<0.001**
**Rural residence**, No. (%), rural	5604 (58.4)	5096 (58.6)	112 (62.2)	148 (57.6)	248 (54.4)	0.44
**Smoking status**, No. (%)						
Never smoker	3698 (38.6)	3343 (38.5)	56 (31.3)	99 (38.5)	200 (44.0)	**0.03**
Former smoker	1355 (14.2)	1149 (13.2)^{c,d}	44 (24.6)^{c,d}	83 (32.3)^{a,b}	79 (17.4)^{a,b}	**<0.001**
Current smoker	4522 (47.2)	4192 (48.3)^{c,d}	79 (44.1)^{c,d}	75 (29.2)^{a,b}	176 (38.7)^{a,b}	**<0.001**
**Smoking exposure**, mean (SD), pack-years	20.4 (26.3)	20.3 (26.1)	27.8 (32.8)	20.5 (26.4)	18.8 (26.2)	0.06
**Hospital admission for severe pulmonary disease in childhood**, No. (%)	381 (3.9)	303 (3.5)^{d}	10 (5.6)^{d}	13 (5.1)^{d}	55 (12.1)^{a,b,c}	**<0.001**
**Indoor to exposure to biomass pollutant**, No. (%)	4739 (49.5)	4266 (49.1)	109 (60.9)	140 (54.5)	224 (49.2)	0.05
**Exposure to dust or chemicals in workplace**, No. (%)	4876 (50.9)	4385 (50.5)	108 (60.0)^{a}	142 (55.3)	241 (52.9)	**0.04**
**Family history of respiratory disease**, No. (%)	3012 (31.4)	2596 (26.6)^{b,c,d}	90 (49.7)^{a,c}	103 (39.4)^{a,b,d}	223 (49.9)^{a,c}	**<0.001**
**BMI**, mean (SD), kg.m^{-2}	24.1 (3.6)	24.1 (3.5)^{b,c,d}	22.9 (3.8)^{a,d}	23.4 (3.8)^{a,d}	24.5 (3.8)^{a,b,c}	**<0.001**
**Obesity**, No. (%)	1242 (12.9)	1119 (11.7)	18 (10.0)	35 (13.6)	70 (15.4)	0.36
**FEV_1 % pred**, mean (SD), %	82.1 (19.8)	83.6 (18.9)^{b,c,d}	60.3 (23.5)^{a,d}	66.4 (22.7)^{a}	70.1 (19.4)^{a,b}	**<0.001**
**FVC % pred**, mean (SD), %	104.2 (19.6)	105.2 (19.2)^{b,c}	88.5 (22.0)^{a,c,d}	93.9 (20.8)^{a,b}	97.9 (19.4)^{b}	**<0.001**
**FEV_1 / FVC**, mean (SD), %	62.5 (8.6)	63.1 (7.9)^{b,c,d}	52.9 (12.8)^{a,d}	54.9 (11.8)^{a}	56.8 (10.3)^{a,b}	**<0.001**
**GOLD Grade**, No. (%)						
GOLD 1	5559 (57.9)	5302 (60.9)^{b,c,d}	38 (21.3)^{a}	77 (30.0)^{a}	142 (31.1)^{a}	**<0.001**
GOLD 2	3369 (35.1)	2945 (33.9)^{d}	72 (40.0)^{d}	110 (42.8)^{d}	242 (53.1)^{a,b,c}	**<0.001**
GOLD 3	587 (6.1)	410 (4.7)^{b,c,d}	54 (30.0)^{a,d}	58 (22.6)^{a}	65 (14.3)^{a,b}	**<0.001**
GOLD 4	78 (0.8)	43 (0.5)^{b,c,d}	16 (8.9)^{a,d}	12 (4.7)^{a,d}	7 (1.5) ^{a,b,c}	**<0.001**
**mMRC Score**, mean (SD)	0.2 (0.6)	0.2 (0.5)^{b,c,d}	1.1 (1.1)^{a,c,d}	0.8 (0.9)^{a,b}	0.6 (0.9)^{a,b}	**<0.001**

Abbreviations: GOLD, Global Initiative for Chronic Obstructive Lung Disease; BMI, body mass index; FEV1, forced expiratory volume in one second; FVC, forced vital capacity; GOLD, Global Initiative for Chronic Obstructive Lung Disease; mMRC, Modified British Medical Research Council Questionnaire.

Note: Data are presented as No. (%) or mean (SD). $^{a, b, c, d}$ showed statistically significant difference between two phenotypes in pairwise comparisons, using Rao-Scott χ^2 test for category variables and Kruskal–Wallis test for continuous variables with Bonferroni-adjusted *P* value.

Bold type represents statistical significance in multiple comparisons.

Supplementary Table 3. Characteristics of COPD patients residing in northern region versus southern region.

	Northern regions	Southern regions	*P* value
**Subjects**	4397 (48·1)	4737 (51·9)	
**Sex** (male)	3072 (69·9)	3563 (75·2)	**0·002**
**Age** (years)	59·8±9·4	62·7±9·6	**<0·001**
**Rural residence** (rural)	2327 (52·9)	2930(61·9)	0·12
**Education Level (**Primary or lower**)**	2217 (50·4)	3000 (63·3)	**<0·001**
**Smoking status**			
Never smoker	1798 (41·0)	1528 (32·3)	**<0·001**
Former smoker	698 (15·9)	747 (15·8)	0·94
Current smoker	1893 (43·1)	2453 (51·9)	**<0·001**
**Smoking exposure (pack-year)**	18·9±25·6	24·9±28·3	**<0·001**
**Hospital admission for severe pulmonary disease in childhood**	161 (3·7)	189 (4·0)	0·45
**Indoor to exposure to biomass pollutant**	2186 (49·8)	2416 (51·1)	0·84
**Exposure to dust or chemicals in workplace**	2059 (46·9)	2491 (52·6)	0·16
**Family history of respiratory disease**	1433 (32·6)	1414 (29·9)	0·21
**BMI (kg.m^{-2})**	24·8±3·6	23·2±3·3	**<0·001**
**CAT Score**	9·9±7·5	9·8±7·0	0·42
**mMRC Score**	0·2±0·6	0·2±0·6	0·87
**Comorbidity number per person**	0·5±0·8	0·4±0·7	**0·04**
**Environmental and social factors**			
Annual average temperature (℃)	11·2 (3·8)	18·4 (2·6)	**<0·001**
Annual average humidity (%)	59·1 (8·4)	77·8 (4·2)	**<0·001**
Annual average $PM_{2·5}$ (μg/m^3)	65·1 (19·2)	48·3 (13·6)	**<0·001**
GDP per capita in 2015 (dollar)	7354 (2910)	7551 (3048)	0·71

Abbreviations: CAT, COPD assessment tool; mMRC, Modified British Medical Research Council Questionnaire; BMI, body mass index; FEV_1, forced expiratory volume in one second; FVC, forced vital capacity; PM, particulate matter; GDP, gross domestic product.

Note: Data are presented as No. (%) or mean±SD. *P* value showed statistically significant difference in comparisons, using Rao-Scott χ^2 test for category variables and Kruskal–Wallis test for continuous variables. Bold type represents statistical significance in comparisons.

Supplementary Table 4. Odds ratio (95% CI) of geographic areas for COPD phenotype with progressive adjustment of risk factors.

	AE with or without CB *vs* NON-AE [a]		ACO *vs* AE and NON-AE [b]	
	Odds ratio (95%CI)	p value	Odds ratio (95%CI)	p value
**Models with geographic areas (South *vs* North)**				
Model 1 [c]	**2·25 (1·72-2·94)**	**<0·001**	**0·72 (0·54-0·96)**	**0·03**
Model 2 [d]	**2·13 (1·63-2·79)**	**<0·001**	0·78 (0·58-1·06)	0·11
Model 3 [e]	**2·10 (1·60-2·76)**	**<0·001**	0·77 (0·57-1·05)	0·10
Model 4 [f]	**2·03 (1·55-2·66)**	**0·01**	**0·75 (0·56-0·99)**	**0·04**
**Model with meteorological factors** [g]				
Temperature (>50th quantile vs <50th quantile)	**1·79 (1·18-2·71)**	**0·006**	**0·61 (0·47-0·78)**	**<0·001**
Humidity (>50th quantile vs <50th quantile)	1·40 (0·70-1·56)	0·85	1·27 (0·97-1·66)	0·09

Abbreviations: AE-CB, exacerbator with chronic bronchitis; AE NON-CB, exacerbator without chronic bronchitis; ACO, asthma-COPD overlap; FEV_1, forced expiratory volume in one second; OR, odds ratio.

Note: Odds ratios with 95% CI were obtained by multiple logistic regressions taking into account complex sample design and cluster effect.

[a] Models were fitted for AE with or without CB phenotype, and NON-AE was taken as reference group.

[b] Models were fitted for ACO phenotype, and AE-CB, AE NON-CB, and NON-AE were taken as reference group.

[c] Model 1: unadjusted.

[d] Model 2: adjusted for age, sex, residential areas and educational level.

[e] Model 3: adjusted for age, sex, residential areas, educational level, and per capita GDP of DSPs in 2015.

[f] Model 4: adjusted for age, sex, residential areas, educational level, per capita GDP of DSPs in 2015, FEV_1 % pred., smoking does, BMI, hospital admission for severe pulmonary disease in childhood, indoor to exposure to biomass for cooking or heating, exposure to dust or chemicals in the workplace, comorbidities, and family history of pulmonary diseases.

[g] Model included average temperature and humidity of DSPs in 2015 instead of geographic areas and were adjusted for those covariates included in model 4.

Bold type represents OR (95% CI) and *P* value with statistical significance in models.

Supplementary Table 5. Characteristics of COPD patients according to GOLD classification based on CAT.

	GOLD categories				p value
	GOLD A (a)	GOLD B (b)	GOLD C (c)	GOLD D (d)	
**Subjects**, No. (%)	4129 (54·1)	3015 (39·5)	127 (1·7)	368 (4·8)	
**Sex**, No. (%), male	3049 (73·8)	2259 (74·9)	86 (67·7)	249 (67·7)[a,b]	0·05
**Age**, mean (SD), years	60·8 (9·6)[b, d]	62·1 (9·5)[a,d]	63·8 (10·3)	64·1 (9·0)[a,b]	**<0·001**
**Rural residence**, No. (%), rural	2257 (54·7)[b]	1921 (63·7)[a,c]	63 (49·6)[b]	215 (58·4)	**0·001**
**Education Level**, No. (%), primary or lower	2250 (54·5)[b,d]	1868 (62·0)[a,c]	72 (56·7)[b]	246 (66·8)[a]	**<0·001**
**Smoking status**, No. (%)					
Never smoker	1459 (35·4)	1018 (33·8)	56 (44·1)	136 (37·1)	0·18
Former smoker	643 (15·6)[c,d]	465 (15·5)[c,d]	31 (24·4)[a,b]	106 (28·9)[a,b]	**<0·001**
Current smoker	2022 (49·0)[c,d]	1526 (50·7)[c,d]	40 (31·5)[a,b]	125 (34·1)[a,b]	**<0·001**
**Smoking exposure**, mean (SD), pack-years	22·1 (26·9)	23·9 (28·3)	18·6 (25·4)	23·0 (29·1)	0·16
**Hospital admission for severe pulmonary disease in childhood**, No. (%)	134 (3·2)[b,c,d]	138 (4·6)[a]	10 (7·9)[a]	25 (6·8)[a]	**<0·001**
**Indoor to exposure to biomass pollutant**, No. (%)	2012 (48·8)[b,d]	1702 (56·5)[a]	60 (47·2)	211 (57·7)[a]	**<0·001**
**Exposure to dust or chemicals in workplace**, No. (%)	2071 (50·2)	1539 (51·1)	65 (51·2)	213 (57·9)	0·31
**Family history of respiratory disease**, No. (%)	1170 (28·3)[b,c,d]	1036 (34·4)[a,d]	52 (40·9)[a]	169 (45·9)[a,b]	**<0·001**
**BMI**, mean (SD), kg.m^{-2}	23·8 (3·4)[d]	23·8 (3·5)[d]	23·4 (3·6)[a,b]	23·2 (3·9)[a,b]	**0·006**
**FEV$_1$**, mean (SD), L	2·3 (0·7)[b,c,d]	2·1 (0·7)[a,c,d]	1·8 (0·7)[a,b,d]	1·5 (0·6)[a,b,c]	**<0·001**
**FEV$_1$ % pred**, mean (SD), %	83·7 (17·5)[b,c,d]	77·6 (21·1)[a,c,d]	72·4 (22·2)[a,b,d]	59·3 (21·3)[a,b,c]	**<0·001**
**FVC**, mean (SD), L	3·6 (0·9)[b,c,d]	3·5 (0·9)[a,c,d]	3·1 (0·9)[a,b,d]	2·9 (0·9)[a,b,c]	**<0·001**
**FVC % pred**, mean (SD) , %	105·2 (18·6)[b,c,d]	101·9 (20·6)[a,c,d]	97·0 (20·3)[a,b,d]	89·3 (20·7)[a,b,c]	**<0·001**
**FEV$_1$ / FVC**, mean (SD), %	62·9 (6·6)[b,c,d]	59·7 (9·3)[a,d]	58·0 (10·2)[a,d]	51·7 (12·1)[a,b,c]	**<0·001**
**CAT Score**, mean (SD)	4·5 (3·7)[b,c,d]	16·0 (5·4)[a,c,d]	5·7 (2·5)[a,b,d]	19·5 (6·4)[a,b,c]	**<0·001**
**mMRC Score**, mean (SD)	0·1 (0·4)[b,c,d]	0·3 (0·7)[a,c,d]	0·6 (0·9)[a,b,d]	1·0 (1·0)[a,b,c]	**<0·001**

Abbreviations: GOLD, Global Initiative for Chronic Obstructive Lung Disease; BMI, body mass index; FEV1, forced expiratory volume in one second; FVC, forced vital capacity; GOLD, Global Initiative for Chronic Obstructive Lung Disease; CAT, COPD assessment tool; mMRC, Modified British Medical Research Council Questionnaire.

Note: Data are presented as No. (%) or mean (SD). [a, b, c, d] showed statistically significant difference between two phenotypes in pairwise comparisons, using Rao-Scott χ^2 test for category variables and Kruskal–Wallis test for continuous variables with Bonferroni-adjusted *P* value.

Bold type represents statistical significance in multiple comparisons.

Supplementary Table 6. Characteristics of COPD patients who were never-smokers.

	Current or ex-Smokers	Never-smokers				
		All	NON-AE	AE-CB	AE NON-CB	ACO
**Subjects**, No. (%)	5791 (63.5)	3326 (36.5)	2977 (89.5)	56 (1.7)	102 (3.1)	191 (5.7) [†]
**Sex**, No. (%), male	5520 (95.3)	1107 (33.3)	1012 (34.0)	22 (39.3)	22 (21.6)	51 (26.7)
**Age**, mean (SD), years	61.5 (9.4)	61.0 (10.0)	61.0 (10.0)	65.6 (9.6)	63.8 (9.7)	58.1 (9.5)
**Hospital admission for severe pulmonary disease in childhood**, No. (%)	182 (3.1)	168 (5.1)	128 (4.3)	3 (5.4)	5 (4.9)	32 (16·8)
**Indoor to exposure to biomass pollutant**, No. (%)	2953 (51.0)	1641 (49.4)	1458 (49.0)	31 (56.4)	53 (52.0)	99 (51.8)
**Exposure to dust or chemicals in workplace**, No. (%)	3154 (54.5)	1391 (41.8)	1229 (41.3)	25 (44.6)	47 (46.1)	90 (47.1)
**Exposure to passive smoking**, No. (%) ⁋	-	1557 (46.8)	1374 (46.2)	19 (33.9)	56 (54.9)	108 (56.5)
**Family history of respiratory disease**, No. (%)	1791 (30.9)	1052 (31.6)	877 (29.5)	26 (46.4)	50 (49.0)	99 (51.8)
**BMI**, mean (SD), kg.m^{-2}	23.6 (3.4)	24.7 (3.6)	24.7 (3.6)	23.9 (4.1)	23.9 (4.0)	24.8 (4.1)

Abbreviations: NON-AE, non-exacerbator; AE-CB, exacerbator with chronic bronchitis; AE NON-CB, exacerbator without chronic bronchitis; ACO, asthma-COPD overlap; GOLD, Global Initiative for Chronic Obstructive Lung Disease; CAT, COPD assessment tool; mMRC, Modified British Medical Research Council Questionnaire.

Note: Data are presented as No. (%).

⁋ Exposure to passive smoking was defined as exposure to passive smoking more than one day in a week during the last 12 months.

† Among 191 ACO patients who were non-smokers, 166 (87%) had self-report exposure to biomass, occupational pollutants, or passive smoking.

Supplementary Table 7. Prevalence of comorbidities in COPD patients according to GOLD classification (CAT).

	GOLD assessment				p value
	GOLD A (a)	GOLD B (b)	GOLD C (c)	GOLD D (d)	
**Comorbidities**	1186 (28·7)^{b,c,d}	1029 (34·1)^{a,d}	57 (44·9)^{a}	164 (44·6)^{a,b}	**<0·001**
Solid tumor	29 (0·7)	22 (0·7)	0 (0)	3 (0·8)	-
Coronary heart disease	187 (4·5)^{b,d}	191 (6·3)^{a,d}	11 (8·7)	55 (14·9)^{a,b}	**<0·001**
Cerebrovascular disease	191 (4·6)^{b,c}	218 (7·2)^{a}	15 (11·8)^{a}	28 (7·6)	**<0·001**
Diabetes mellitus	187 (4·5)	169 (5·6)	7 (5·5)	21 (5·7)	0·26
Hypertension	819 (19·8)	670 (22·2)	37 (29·1)	91 (24·7)	**0·007**
Depression	17 (0·4)^{c}	17 (0·6)	3 (2·4)^{a}	2 (0·5)	**0·02**
Osteoporosis	145 (3·5)^{d}	142 (4·7)^{d}	8 (6·3)	36 (9·8)^{a,b}	**<0·001**
**Number of Comorbidities**					
0	2942 (71·3)^{b,c,d}	1985 (65·9)^{a,d}	70 (55·1)^{a}	204 (55·4)^{a,b}	**<0·001**
1-2	1117 (27·1)^{b,c,d}	949 (31·5)^{a,d}	52 (40·9)^{a}	142 (38·6)^{a,b}	**<0·001**
≥3	69 (1·7)^{d}	80 (2·7)^{d}	5 (3·9)	22 (6·0)^{a,b}	**<0·001**

Abbreviations: GOLD, Global Initiative for Chronic Obstructive Lung Disease.

Note: Data are presented as No. (%). $^{a, b, c, d}$ showed statistically significant difference between two phenotypes in pairwise comparisons, tested by Rao-Scott χ^2 test using Bonferroni-adjusted *P* value. Bold type represents statistical significance in multiple comparisons.

Supplementary Table 8. Treatment and prevention in COPD patients according to GOLD classification (CAT).

	GOLD assessment				p value
	GOLD A (a)	GOLD B (b)	GOLD C (c)	GOLD D (d)	
**Therapy**					
**Inhaled therapy**	46 (1·1)[b,c,d]	94 (3·1)[a,c,d]	34 (26·8)[a,b]	116 (31·5)[a,b]	**<0·001**
Mono-ICS	0 (0)	3 (0·1)[c]	1 (1·6)[b]	3 (0·8)	NA
Short-acting bronchodilators	17 (0·4)[b,c,d]	26 (0·9)[a,c,d]	5 (3·9)[a,b]	28 (7·6)[a,b]	**<0·001**
Long-acting bronchodilators	7 (0·2)[d]	9 (0·3)[d]	1 (0·8)	10 (2·7)[a,b]	**<0·001**
Combined therapy [e]	1 (0·02)[b,c,d]	15 (0·5)[a,d]	2 (1·6)[a]	15 (4·1)[a,b]	**<0·001**
Combined- ICS [e]	0 (0)	7 (0·2)[d]	1 (1·6)	9 (2·7)[b]	NA
Unknown inhaler therapy [f]	21 (0·5)[b,c,d]	41 (1·4)[a,c,d]	24 (18·9)[a,b]	60 (16·3)[a,b]	**<0·001**
**Non-inhaled therapy**	171 (4·1)[b,c,d]	373 (12·4)[a,c,d]	80 (63·0)[a,b]	245 (66·6)[a,b]	**<0·001**
Theophylline	41 (1·0)[b,c,d]	128 (4·2)[a,c,d]	13 (10·2)[a,b,d]	78 (21·2)[a,b,c]	**<0·001**
Oral corticosteroids	7 (0·2)[c,d]	13 (0·4)[c,d]	5 (3·9)[a,b]	17 (4·6)[a,b]	**<0·001**
Expectorant	23 (0·6)[b,c,d]	72 (2·4)[a,c,d]	20 (15·7)[a,b]	59 (16·0)[a,b]	**<0·001**
Antioxidants	1 (0·02)[c]	7 (0·2)[c]	2 (1·6)[a,b]	1 (0·3)	**<0·001**
Cough medicine	85 (2·1)[b,c,d]	191 (6·3)[a,c,d]	33 (26·0)[a,b]	135 (36·7)[a,b]	**<0·001**
Antibiotics	83 (2·0)[b,c,d]	206 (6·8)[a,c,d]	34 (26·8)[a,b]	140 (38·0)[a,b]	**<0·001**
Traditional Chinese medicine	48 (1·2)[b,c,d]	127 (4·2)[a,c,d]	22 (17·3)[a,b]	97 (26·4)[a,b]	**<0·001**
Antiallergic medicine	8 (0·2)[b,c,d]	21 (0·7)[a,c,d]	5 (3·9)[a,b]	27 (7·3)[a,b]	**<0·001**
**PFT done before**	214 (5·2)[c,d]	171 (5·7)[c,d]	19 (15·0)[a,b]	70 (19·0)[a,b]	**<0·001**
**Rehabilitation**	20 (0·5)[c,d]	20 (0·7)[c,d]	6 (4·7)[a,b]	24 (6·5)[a,b]	**<0·001**
**Oxygen therapy**	23 (0·6)[b,c,d]	55 (1·8)[a,c,d]	23 (18·1)[a,b,d]	113 (30·7)[a,b,c]	**<0·001**
**Influenza vaccine**	119 (2·9)[d]	114 (3·8)	7 (5·5)	20 (5·4)[a]	**0·02**
**Pneumococcal vaccine**	31 (0·8)[c]	21 (0·7)[c]	4 (3·1)[a,b]	7 (1·9)	**0·001**

Abbreviations: GOLD, Global Initiative for Chronic Obstructive Lung Disease; ICS, inhaled corticosteroid; PFT, pulmonary function testing.

Note: Data are presented as No. (%). [a, b, c, d] showed statistically significant difference between two phenotypes in pairwise comparisons, using Rao-Scott χ^2 test with Bonferroni-adjusted p value.

[e] Combined ICS was contained in the combined therapy.

[f] Patients do not know or remember the type of inhaled medications.

Bold type represents statistical significance in multiple comparisons.
