# Supplementary material for: Phenotype and management of chronic obstructive pulmonary disease patients in general population in China: a nationally cross-sectional study
Source: NPJ Prim Care Respir Med. 2021 Jun 1;31:32. doi: 10.1038/s41533-021-00243-x (PMC8169915; doi:10.1038/s41533-021-00243-x)
Supplement: Supplementary file 2 — Reporting Summary [file 41533_2021_243_MOESM2_ESM.pdf]

nature research

Corresponding author(s): Liwen Fang

Last updated by author(s): 26 April 2021

Reporting Summary

Nature Research wishes to improve the reproducibility of the work that we publish. This form provides structure for consistency and transparency in reporting. For further information on Nature Research policies, see our Editorial Policies and the Editorial Policy Checklist.

Statistics

For all statistical analyses, confirm that the following items are present in the figure legend, table legend, main text, or Methods section.

n/a	Confirmed	
☐	☒	The exact sample size (n) for each experimental group/condition, given as a discrete number and unit of measurement
☒	☐	A statement on whether measurements were taken from distinct samples or whether the same sample was measured repeatedly
☐	☒	The statistical test(s) used AND whether they are one- or two-sided<br>*Only common tests should be described solely by name; describe more complex techniques in the Methods section.*
☐	☒	A description of all covariates tested
☐	☒	A description of any assumptions or corrections, such as tests of normality and adjustment for multiple comparisons
☐	☒	A full description of the statistical parameters including central tendency (e.g. means) or other basic estimates (e.g. regression coefficient) AND variation (e.g. standard deviation) or associated estimates of uncertainty (e.g. confidence intervals)
☐	☒	For null hypothesis testing, the test statistic (e.g. F, t, r) with confidence intervals, effect sizes, degrees of freedom and P value noted<br>*Give P values as exact values whenever suitable.*
☒	☐	For Bayesian analysis, information on the choice of priors and Markov chain Monte Carlo settings
☐	☒	For hierarchical and complex designs, identification of the appropriate level for tests and full reporting of outcomes
☒	☐	Estimates of effect sizes (e.g. Cohen's d, Pearson's r), indicating how they were calculated

Our web collection on statistics for biologists contains articles on many of the points above.

Software and code

Policy information about availability of computer code

Data collection	No software was used.
Data analysis	All analyses were performed by using SAS 9.4 version and R 3.5.4.

For manuscripts utilizing custom algorithms or software that are central to the research but not yet described in published literature, software must be made available to editors and reviewers. We strongly encourage code deposition in a community repository (e.g. GitHub). See the Nature Research guidelines for submitting code & software for further information.

Data

Policy information about availability of data

All manuscripts must include a data availability statement. This statement should provide the following information, where applicable:
- Accession codes, unique identifiers, or web links for publicly available datasets
- A list of figures that have associated raw data
- A description of any restrictions on data availability

All data that support the findings of this study are included in this published article and its supplementary information files. The datasets are available on request from the corresponding author.

Field-specific reporting

Please select the one below that is the best fit for your research. If you are not sure, read the appropriate sections before making your selection.

☐ Life sciences ☒ Behavioural & social sciences ☐ Ecological, evolutionary & environmental sciences

For a reference copy of the document with all sections, see nature.com/documents/nr-reporting-summary-flat.pdf

Behavioural & social sciences study design

All studies must disclose on these points even when the disclosure is negative.

Study description	a quantitative cross-sectional study
Research sample	A total of 75,107 adults aged 40 years or older were interviewed in a cross-sectional study, and 2,282 individuals were not eligible for spirometry. Prebronchodilator and post-bronchodilator examinations were completed by 68,984 people. In total, 66,752 participants took part in the interview and had acceptable post-bronchodilator spirometry examinations. Finally, we identified 9,134 participants as COPD patients. Most of patients were male (72.6%), and the mean age was 61.3 years.
Sampling strategy	The national chronic obstructive pulmonary diseases (COPD) prevalence study was a nationwide cross-sectional study during 2014-2015, using the integrated national disease surveillance point (DSP) system from the Chinese Center for Disease Control and Prevention to obtain a nationally representative sample of the general population in China. In this study, we used a complex, multistage, probability sampling strategy, and included Chinese citizens aged 40 years or older who had been living in their current residence for at least 6 months within the year before the survey, to exclude new immigrants. The overall response rate, using the standard definition by the American Association for Public Opinion Research, was 96.3%.
Data collection	We invited all people who fulfilled the inclusion criteria to an interview. Trained staff from local health stations or community clinics administered a comprehensive questionnaire to obtain information on demographic characteristics, medical history, COPD specific risk factors, and respiratory symptoms. Trained staff did spirometry on all participants who were eligible for the procedure, using the same brand of spirometer (MasterScreen Pneumo, Jaeger, Germany). Spirometry was performed following recommendations by the American Thoracic Society. We measured both prebronchodilator and post-bronchodilator forced vital capacity (FVC), FEV1, forced expiratory volume in 6 s (FEV6), and peak expiratory flow. We used GOLD lung function criteria to define individuals with COPD—i.e., those with a post-bronchodilator FEV1:FVC less than 70%. We did further testing of individuals with COPD, consisted of the modified Medical Research Council (mMRC) dyspnea score and the Global Initiative for Chronic Obstructive Lung Disease (GOLD) 2017 ABCD assessment tool.
Timing	From December 2014 to December 2015.
Data exclusions	We exclude participants who were ineligible for spirometry due to severe diseases and those who had a post-bronchodilator FEV1/FVC>0.7.
Non-participation	8355 participants were excluded due to invalid spirometry and 57,618 participants who had normal spirometry (post-bronchodilator FEV1/FVC>0.7) were excluded from this study.
Randomization	Not applicable.

Reporting for specific materials, systems and methods

We require information from authors about some types of materials, experimental systems and methods used in many studies. Here, indicate whether each material, system or method listed is relevant to your study. If you are not sure if a list item applies to your research, read the appropriate section before selecting a response.

Materials & experimental systems

n/a	Involved in the study	
☒	☐	Antibodies
☒	☐	Eukaryotic cell lines
☒	☐	Palaeontology and archaeology
☒	☐	Animals and other organisms
☐	☒	Human research participants
☒	☐	Clinical data
☒	☐	Dual use research of concern

Methods

n/a	Involved in the study	
☒	☐	ChIP-seq
☒	☐	Flow cytometry
☒	☐	MRI-based neuroimaging

Human research participants

Policy information about studies involving human research participants

Population characteristics	Of the 75,107 participants in the survey, 8,355 participants were excluded due to severe diseases or ineligible spirometry, and 57,618 with a FEV1/FVC ≥0.7 were excluded. Finally, the study detected 9,134 spirometry-confirmed COPD patients aged ≥40 years, of whom all were available for classification of predefined phenotype and 7,639 for GOLD classification. The mean age was 61.3 years (SD 9.6), 3,326 were never-smokers, 246 were ever diagnosed as asthma, and 1,132 patients were obesity.
Recruitment	The study used a complex, multistage, probability sampling strategy, and included Chinese citizens aged 40 years or older who had been living in their current residence for at least 6 months within the year before the survey, to exclude new immigrants. We invited all sampled participants to take part in the study and complete an interview. In this study, we included all COPD patients who were identified from the sampling in the analysis.
Ethics oversight	The study protocol was approved by the Ethical Review Committee of the National Center for Chronic and Non-communicable Disease Control and Prevention, China CDC. Written informed consent was obtained from all study participants (NO. 201410).

Note that full information on the approval of the study protocol must also be provided in the manuscript.
